# Effect of SiO_2_/Al_2_O_3_ ratio on the electrochemical performance of amorphous zeolite loaded with cobalt oxide grown *via* steam-assisted crystallization method

**DOI:** 10.1039/d3ra03268j

**Published:** 2023-07-17

**Authors:** Saureille Ngouana Moafor, Patrice Kenfack Tsobnang, Kabir Oyeniran Oyedotun, Roussin Lontio Fomekong, Guy L. Kabongo, Macheli Lebohang, John Ngolui Lambi, Linda L. Jewell

**Affiliations:** a Department of Chemical Engineering, University of South Africa (UNISA) Christiaan De Wet & Pioneer Avenue Florida 1710 South Africa saureilemoafor@gmail.com jewelll@unisa.ac.za; b Laboratory of Material Chemistry, Department of Inorganic Chemistry, University of Yaoundé I (UYI) P. O. Box 812 Yaoundé Cameroon; c Laboratory of Solid State and Molecular Inorganic Chemistry, Department of Chemistry, University of Dschang Cameroon; d College of Science, Engineering and Technology (CSET), University of South Africa Florida Campus Johannesburg 1710 South Africa; e Department of Physics, College of Science, Engineering and Technology, University of South Africa Johannesburg 1710 South Africa

## Abstract

Improving the performance of a supercapacitor is one of the main approaches to solve the energy shortage problem. Electrode material is one of the key components limiting the efficiency of a supercapacitor. Discovering, tuning, and improving electrode materials are very important. This work reports the effect of SiO_2_/Al_2_O_3_ ratio on electrochemical performances of amorphous zeolites ZSM5 (AZ) and H-ZSM5 (H-AZ) loaded with cobalt oxide. Two SiO_2_/Al_2_O_3_ ratios (1 = 6.2 and 2 = 8.3) of AZ_1_, AZ_2_ and H-AZ_1_, H-AZ_2_ were synthesized by a facile impregnation method. Then, controlled masses of cobalt oxide were introduced to enhance the supercapacitive performances of the amorphous zeolite. Investigation of the SiO_2_/Al_2_O_3_ ratio in the cobalt oxide/zeolite composite (Co/AZ and Co/H-AZ) was carried out to unveil its effect on the electrochemical properties. Worthy of note is the fact that the resulting electrode materials exhibited supercapacitive behavior that is effective over a potential window ranging from 0 to 0.5 V in potassium hydroxide (1 M KOH) aqueous electrolyte. Results from Galvanometry Charging and Discharging (GCD) analyses show that the modified Ni-foam electrodes loaded with Co/H-AZ_1_ and Co/H-AZ_2_ are capable of delivering a relatively high specific capacity from 45.97 mA h g^−1^ to a high value of 72.5 mA h g^−1^ at 1 A g^−1^ and Ni-foam electrodes loaded with Co/AZ_1_ and Co/AZ_2_ exhibited values from 26 mA h g^−1^ to 52.83 mA h g^−1^ respectively. It is clearly shown that, when the mass ratio SiO_2_/Al_2_O_3_ increases, the specific capacity increases as well. It was also noticed that after 2000 cycles, Co/H-AZ_1_ and Co/AZ_1_ have a poor coulombic efficiency while Co/H-AZ_2_ and Co/AZ_2_ exhibited 98% for coulombic efficiency. Finally, this study shows that to fabricate high performance supercapacitors with amorphous zeolite loaded with cobalt oxide, one should keep the ratio of SiO_2_/Al_2_O_3_ as high as possible during synthesis.

## Abstract 2

The investigation of SiO_2_/Al_2_O_3_ ratio on electrochemical performance of amorphous zeolite loaded with cobalt oxide reveals that it is important to keep that ratio as high as possible for optimum supercapacitive properties.

## Graphical abstract

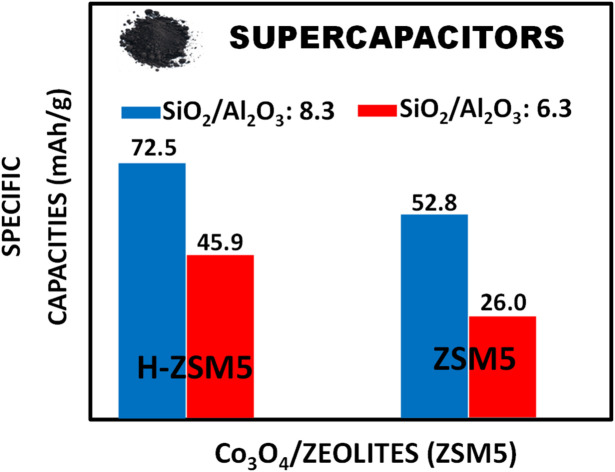

## 1. Introduction

The scientific community is increasingly attracted by electrochemistry and more precisely by supercapacitors in recent years.^1^ Supercapacitors are characterized by their capability of delivering high power, fast charge and discharge rates, and long cycle life required for power devices.^2^ It is one of the possible solutions to provide energy in rural areas, where there is no electricity. Some portable common electronic devices like smart phones, laptops, *etc.* can also use supercapacitors as a power bank. Among active materials for the fabrication of electrodes for supercapacitors, transition metal oxides, working mostly as pseudocapacitors,^3^ are a very good choice.^4^ The most popular are Co_3_O_4_,^5^ RuO_2_,^6^ MnO_2_,^3^ NiO,^7^ and MoO_2_ because of their environmental affability, easy accessibility and higher energy density.^8^ Among all these, Co_3_O_4_ has attracted a particular interest because of its high theoretical specific potential and good electrochemical capability.^9,10^ However, Co_3_O_4_ has a low inherent conductivity, a limited potential or energy density and small cycling stability. For example Indira *et al.* obtained 500 cycles while with other electrode materials, 2000 to 5000 cycles can easily be reached.^11^ These limitation for Co_3_O_4_ can be overcome by preparing composite-based cobalt oxide materials. In recent years, Co_3_O_4_ has been coupled with rGO, Ni(OH)_2_, NiO, ZnO, *etc.* for high performance electrode material for supercapacitor.^12,13^ Although great progress has been made on Co_3_O_4_ based composite materials, it is still very important to develop composite electrodes comprising Co_3_O_4_ as the most favorable candidates for high performance supercapacitor electrodes.

Zeolites have played a very important role in the chemical and petroleum industries for many years. Zeolites are known as a unique tetrahedral structured material arranged in connected rings forming micropores which offer a high surface area and high ion exchange capacity. Based on that and its ability to increase its porosity, zeolites can be used as a matrix in order to enhance the specific capacitance or specific capacity of metal oxides.^14^ It is important to mention that previous reports have demonstrated that zeolite alone is not a good candidate as electrode materials for supercapacitor.^15^

It should also be noted that zeolites can be categorized in relation to the Si/Al ratio, *i.e.* low silica zeolites (Si/Al mole ratio = 1–2, SiO_2_/Al_2_O_3_ mass ratio = 1.18–2.35), medium silica zeolites (Si/Al mole ratio = 3–10, SiO_2_/Al_2_O_3_ mass ratio = 3.53–11.76) and high silica zeolites (Si/Al mole ratio = 10–∞, SiO_2_/Al_2_O_3_ mass ratio = 11.76–∞).^16^ So, the physical and chemical properties of zeolites vary according to the SiO_2_/Al_2_O_3_ ratio.^17^ A synthesized zeolite from silica and aluminium essentially provides acidic sites.^18^ The study of the impact of the Si/Al ratio is applied in several fields of science. For example, for zeolite, Y (FAU), the higher the SiO_2_/Al_2_O_3_ ratio is, the more stable is its structure, but it is limited in terms of thermal and hydrothermal stability. It is also the most widely used zeolite in oil refineries due to its large pores and its surface area.^19^ Therefore, a thermally stable Y, called ultra-stable Y zeolite (USY), was developed by dealumination of the frame by controlled steam supply.^20^ The reduced ion exchange capacity and a smaller unit cell are indications of the removal of aluminium from the framework.^21,22^ Fuat *et al.* established the effects of zeolite framework structure and Si/Al ratio on the gas phase carboxylation of DMM to MMAc and found that the effects of zeolite framework structure and SiO_2_/Al_2_O_3_ ratio are interpreted in the light of a proposed reaction mechanism.^23^ Patrick J. *et al.* tested the hypothesis that SiO_2_/Al_2_O_3_ mass ratio was responsible for the systematic variation in EPR parameters for group 1 and 2 zeolites. For this purpose, a series of copper-exchanged zeolites with different SiO_2_/Al_2_O_3_ ratios were prepared.

In this work, for the first time, to the best of our knowledge, synthetised amorphous zeolite ZSM5 (named AZ) and HZSM5 (named H-AZ) materials with 2 SiO_2_/Al_2_O_3_ mass ratios are used to prepare amorphous zeolite loaded with cobalt oxide (named Co/AZ and Co/H-AZ) as new electrode material for supercapacitor. Moreover, the effect of SiO_2_/Al_2_O_3_ mass ratio on electrochemical properties of the prepared composite material was also investigated. The results obtained indicate that the high value of SiO_2_/Al_2_O_3_ mass ratio is beneficial for electrochemical properties in pristine and composite based zeolite material.

## 2. Experimental section

### 2.1 Chemicals

Tetraethyl-orthosilicate (TEOS, 98%, Aldrich), aluminium hydroxide (Al(OH)_3_, 99.6%, Aldrich), tetra propylammonium bromide (TPABr, 99%, Aldrich), ammonium nitrate (98%, Sigma-Aldrich), ethanol (98%, Merck) and cobalt nitrate hexahydrate (99%, Aldrich) were used without further purification.

### 2.2 Co/AZ and Co/H-AZ preparation at 2 mass ratio SiO_2_/Al_2_O_3_

**Scheme 1:**
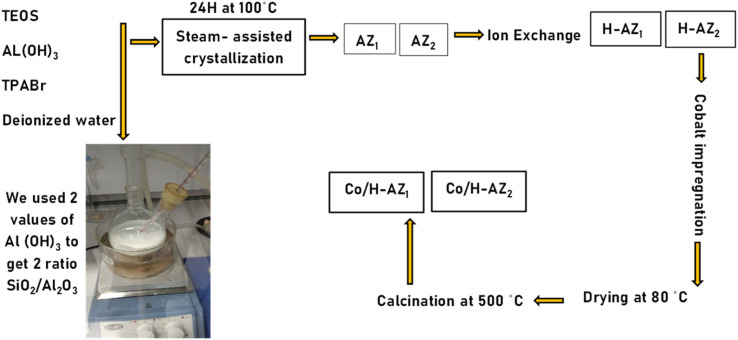
Protocol for amorphous zeolite loaded with cobalt oxide synthesis at 2 ratios.

2 mass ratios SiO_2_/Al_2_O_3_ of mesoporous zeolites ZSM5 and H-ZSM5 were synthesized by the steam-assisted crystallization method (SAC) (see Scheme 1).^24^ For the first mass ratio (SiO_2_/Al_2_O_3_: 6.2), 11.32 g of tetra propylammonium bromide (TPABr) and 0.3275 g of aluminium hydroxide (Al(OH)_3_) were first added in sequence into 100 mL H_2_O, followed by dropping 27 mL of tetraethyl orthosilicate (TEOS) and the mixture was stirred for 5 h at 60 °C. The mixture was then transferred in a Teflon bottle into an autoclave, aged for 12 h at 80 °C and finally steam treated at 100 °C for 12 h. The precipitate obtained (called AZ_1_) was washed with ethanol, dried at room temperature, and calcined at 300 °C in a furnace for 4 h. A second reaction was performed under the same synthesis conditions except that the mass of Al(OH)_3_ was 0.2275 g (mass ratio 2 SiO_2_/Al_2_O_3_: 8.3) and gave zeolite AZ_2_. An ion exchange process was performed using a 1 M solution of NH_4_NO_3_ and AZ_1_, AZ_2_ at 60 °C for 2 h. After this process, the materials obtained were filtered and washed with de-ionized water and further dried in an oven at 60 °C overnight. The resulting NH_4_–AZ_1_ and NH_4_–AZ_2_ were calcined in a furnace at 300 °C for 3 h to obtain H-AZ_1_ and H-AZ_2_. Cobalt nitrate (1.766 g) was dissolved in the required volume of deionized water (1.5 mL) to obtain 15 wt% Co solution to impregnate the supports AZ_1_, AZ_2_ and H-AZ_1_, H-AZ_2_ (2 g of zeolite) with a mass-loading of 85%. The obtained catalysts were then dried in an oven at 60 °C for 6 h and calcined at 400 °C for 4 h. The obtained materials were named Co/AZ_1_, Co/AZ_2_ and Co/H-AZ_1_, Co/H-AZ_2_ respectively.

### 2.3 Materials characterization

X-ray diffraction (XRD) analysis was employed for the phase identification of the catalysts. The powder XRD machine (Rigaku Smart lab diffractometer) which was equipped with a monochromatic Cu Kα radiation (*λ* = 1.54 Å) source was operated at 200 mA and 45 kV. The morphologies and microstructures of the as-prepared materials were analysed by scanning electron microscopy (SEM) using a JSM-7100 L, JEOL instrument. The surface area of the materials was analysed using the N_2_ adsorption–desorption Brunauer–Emmett–Teller (BET) method, while pore size distribution was determined using the Barrett–Joyner–Halenda (BJH) method on the Tristar II instrument. Fourier transform infra-red (FTIR) analyses, carried out on a Bruker FTIR spectrometer (Vertex 70 model), provided more information on the chemical structure.

### 2.4 Electrochemical measurements

For the electrochemical measurements, the working electrodes were prepared by mixing each sample (Co/AZ_1_, Co/AZ_2_ and Co/H-AZ_1_, Co/H-AZ_2_) with carbon black mesoporous and polyvinylidene fluoride (PVDF) in a ratio of 16 : 2 : 2 into dimethyl sulfoxide (DMSO). The slurries, obtained with the assistance of ultrasonication for 15 min, were then drop-cast onto 1 × 1 cm pieces of nickel foam previously washed with a solution of 8.3 mL hydrochloric acid and 50 mL of distilled water. The electrodes so prepared were dried at 110 °C for 1 h and the electrochemical data was subsequently collected on an Auto lab PGSTAT302N Potentiostat using a three-electrode system. Platinum wire and Ag/AgCl (3 M KCl-filled) were used as counter and reference electrodes, respectively. A 1 M KOH solution served as the electrolyte. Electrochemical Impedance Spectroscopy (EIS) measurements were performed with an AC amplitude of 5 mV in the frequency range of 100 kHz to 100 mHz. Finally, Linear Sweep Voltammetry (LSV) was also performed.

## 3. Results and discussion

### 3.1 XRD analysis

**Fig. 1:**
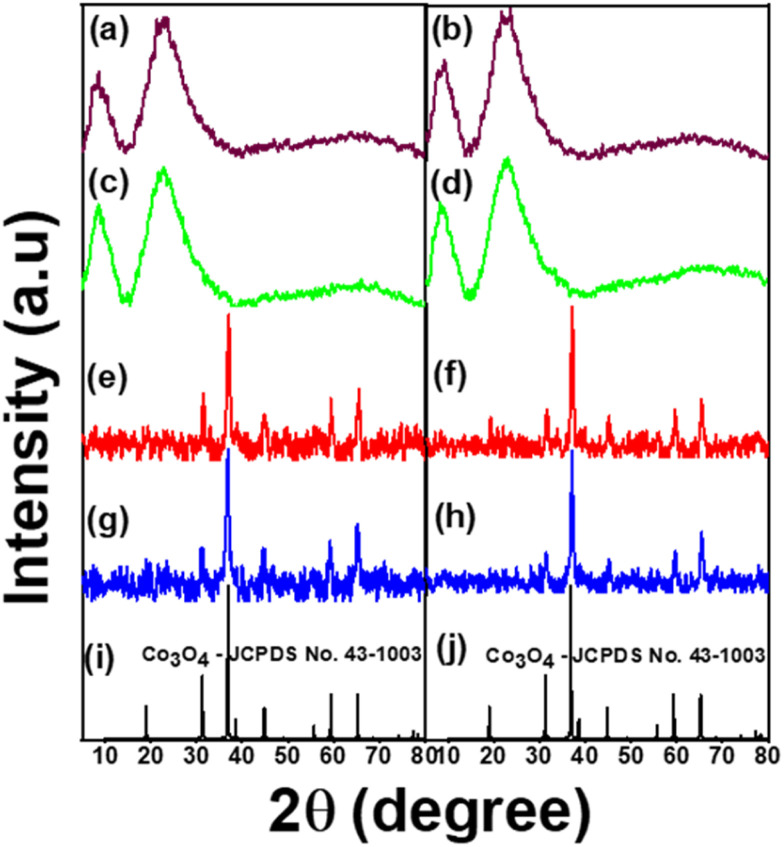
XRD pattens of (a) AZ_1_, (b) AZ_2_, (c) H-AZ_1_, (d) H-AZ_2_, (e) Co/AZ_1_, (f) Co/AZ_2_, (g) Co/H-AZ_1_, (h) Co/H-AZ_2_, (i and j) Co_3_O_4_-JCPDS no. 43-1003.

The XRD patterns of all the samples are depicted in Fig. 1. The results show that AZ_1_, AZ_2_ and H-AZ_1_, H-AZ_2_ exhibited two broad peaks at 2*θ* positions of 8° and 23° for AZ_1_, H-AZ_1_ and 9° and 22° for AZ_2_, H-AZ_2_ respectively. This confirms that amorphous zeolite ZSM5 has been successfully obtained, which is in agreement with previous reports.^15,25,26^. Upon incorporating cobalt, it was noticed that the zeolite peaks intensity decreased considerably, indicating that the cobalt oxide has certainly covered the zeolite surface. The data shows variable Co oxidation states that are influenced by Co incorporation and Co particle sizes.^27^ Co/AZ_1_ and Co/H-AZ_1_ (Co/AZ_2_ and Co/H-AZ_2_) are not similar, which implies that H^+^ ions affected the XRD of Co/H-AZ_1_ and Co/H-AZ_2_. We also have Co_3_O_4_ peaks on Co/AZ_1_, Co/AZ_2_ and Co/H-AZ_1_, Co/H-AZ_2_. Hence, it was obvious that the zeolite materials synthesized with different SiO_2_/Al_2_O_3_ mass ratios are not very similar.^28^ The diffraction peaks at 31.54°, 37.19°, 56° and 65° which correspond to (2 2 0), (3 1 1), (5 1 1) and (4 4 0) lattice planes, respectively, for Co_3_O_4_ indicate that cobalt zeolite composite was successfully obtained.^29,30^ The structure obtained was indexed to JCPDS no. 43-1003 card number, which confirmed its cubic crystalline nature.^31^

### 3.2 FTIR analysis

**Fig. 2:**
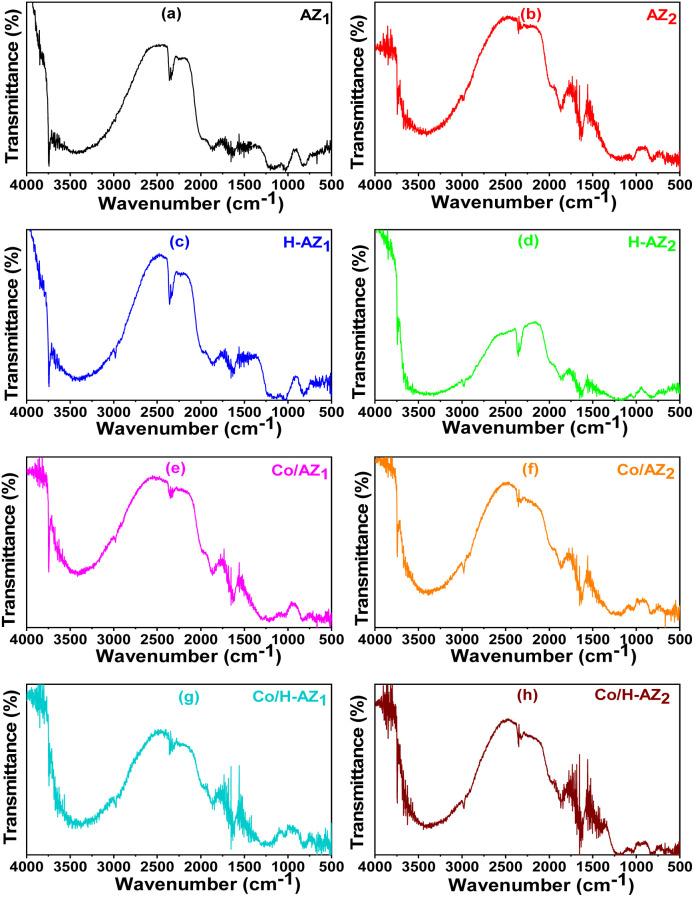
FTIR patterns for (a) AZ_1_, (b) AZ_2_, (c) H-AZ_1_, (d) H-AZ_2_, (e) Co/AZ_1_, (f) Co/AZ_2_, (g) Co/H-AZ_1_, (h) Co/H-AZ_2_.

FT-IR measurements were performed to elucidate the chemical bonds in the prepared samples. The spectra of all the samples are presented in Fig. 2. It was observed that all the samples exhibited a wideband between 3000 and 3750 cm^−1^, which corresponds to the stretching vibration of the hydroxyl (OH) group due to adsorbed water and the Si–OH and Al–OH bonds of the HZSM-5 surface groups.^32^ So, it can be noticed that the mass ratio SiO_2_/Al_2_O_3_ did not affect much the bonds. The peaks at 2351 cm^−1^ and 2991 cm^−1^ can be associated with the stretching vibrations of the C–H bond on the 1-heptyl group. The peak around 1612 cm^−1^ is attributed to the bending mode of the OH of water absorbed in the channels of the zeolite while the peaks at 1218 cm^−1^ and 1451 cm^−1^ can be associated with the vibration of the imidazole ring.^33,34^ The band at around 950–1090 cm^−1^ is due to the asymmetric stretching mode of Si–O–T (T = Si or Al).^35,36^ The vibrational frequencies at about 502, 560, 809, 1034–1292 cm^−1^ are characteristic of MFI type zeolites.^37^

### 3.3 SEM analysis

**Fig. 3:**
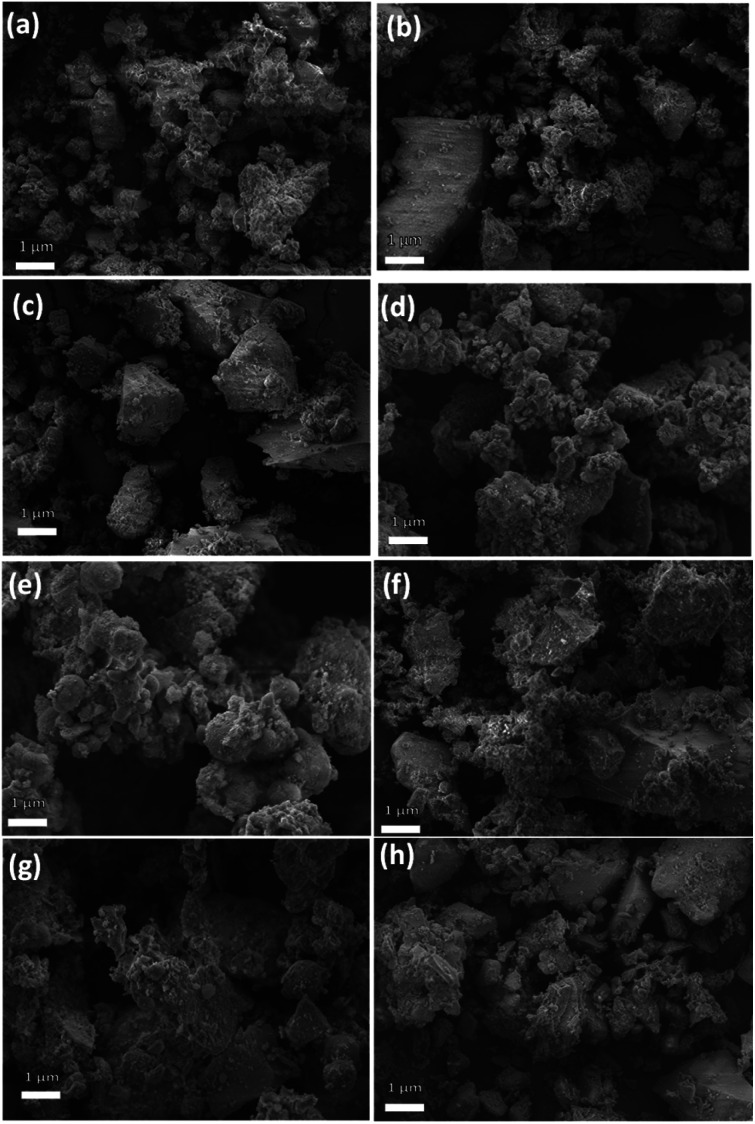
SEM images of (a and b) AZ_1_ and AZ_2_ (c and d) H-AZ_1_ and H-AZ_2_, (e and f) Co/AZ_1_ and Co/AZ_2_, (g and h) Co/H-AZ_1_ and Co/H-AZ_2_.

**Fig. 4:**
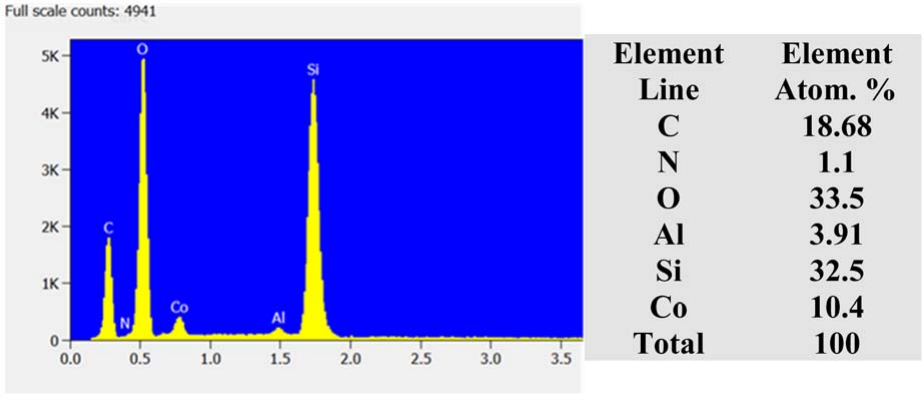
EDS and elemental composition of Co/H-AZ_2_.

The microstructural analysis of the zeolite composites was further examined by HRSEM. The SEM micrographs (Fig. 3) reveal that AZ_1_, AZ_2_ and H-AZ_1_, H-AZ_2_ exhibit relatively amorphous particles formed by agglomerates of nano-sized crystals.^38^ Moreover, it can be noticed that when SiO_2_/Al_2_O_3_ mass ratio increases, the same happened for the agglomeration.^39^ Interestingly, Fig. 3e–h show clearly that the impregnation with cobalt considerably increased the agglomeration of the zeolites. Nevertheless, after adding cobalt on H-AZ_1_ and H-AZ_2_ (Fig. 3g and h), the crystalline shape remained almost the same with different SiO_2_/Al_2_O_3_ mass ratios, which is in agreement with the XRD results.^40^ EDS and elemental composition of Co/H-AZ_2_ are shown in Fig. 4 and the results indicate that the expected composition was obtained.

### 3.4 N_2_ adsorption/desorption analysis

**Fig. 5:**
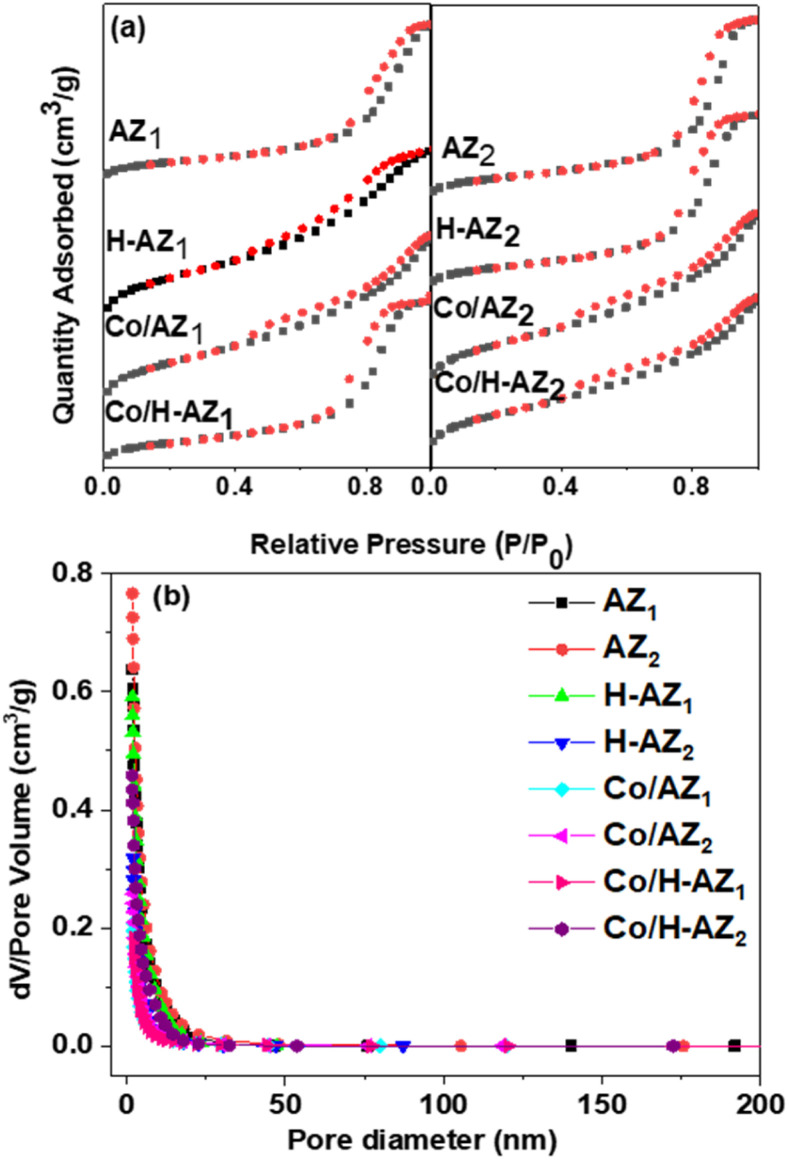
(a) N_2_-sorption isotherms (b) pore size distribution.

Nitrogen adsorption–desorption measurements were carried out using BET. The results of the pore structures and surface areas of AZ_1_, AZ_2_ and H-AZ_1_, H-AZ_2_, Co/AZ_1_, Co/AZ_2_ and Co/H-AZ_1_, Co/H-AZ_2_ are displayed in Fig. 5. The results (Fig. 5a) show clearly defined hysteresis loops typical of type IV isotherms (based on the IUPAC classification), thus, revealing a mesoporous structure for the zeolites.^41,42^ It can be noticed that all the samples that contained H^+^ had their isotherm shape changed when the mass ratio SiO_2_/Al_2_O_3_ increased while still belonging to type IV isotherms. Fig. 5b shows the pore diameter distribution for all the samples, while the textural properties of the samples are displayed in Table 1. The BJH pore size distribution for all the samples (Fig. 5b) shows a narrow pore size distribution not centered alongside a wide pore size distribution. The main effect of the sample preparation is an increase in the pore sizes in the range of 20 nm.^43^ This confirms that all the samples are characteristically mesoporous materials and the mass ratio SiO_2_/Al_2_O_3_ did not affect it. The mesopores for the samples show a pore size distribution in the range 5.45–11.56 nm, which is also characteristic of mesoporous materials.

**Table 1:** Textual properties of AZ_1_, AZ_2_, H-AZ_1_, H-AZ_2_, Co/AZ_1_, Co/AZ_2_, Co/H-AZ_1_, Co/H-AZ_2_

	Bet surface are^a^a (m^2^ g^−1^)	Micropore area^b^ (m^2^ g^−1^)	External area^c^ (m^2^ g^−1^)	Pore diameter^d^ (nm)	Pore volume^e^ (cm^3^ g^−1^)	Co_3_O_4_ size (nm) from XRD
AZ_1_	458.05	—	464.71	10.81	1.39	—
AZ_2_	490.87	—	595	2.52	0.26	—
H-AZ_1_	366.97	—	373.61	9.93	1.05	—
H-AZ_2_	390.19	14.06	376	5.2	0.5	—
Co/AZ_1_	275.84	—	282.35	9.6	0.79	6.54
Co/AZ_2_	348.32	—	355.98	7.5	0.72	6.61
Co/H-AZ_1_	254.62	—	268.59	6.2	0.45	6.49
Co/H-AZ_2_	298.98	—	302.20	9.95	0.86	6.69

a Surface area.

b Microporous surface area.

c External surface area.

d Pore diameter.

e Pore volume.

The specific surfaces area of all the samples are presented in Table 1 indicating that when the mass ratio SiO_2_/Al_2_O_3_ increased, the surface area also increased, which is an important indication that the material would be very good for electrochemical applications.

### 3.5 XPS analysis

**Fig. 6:**
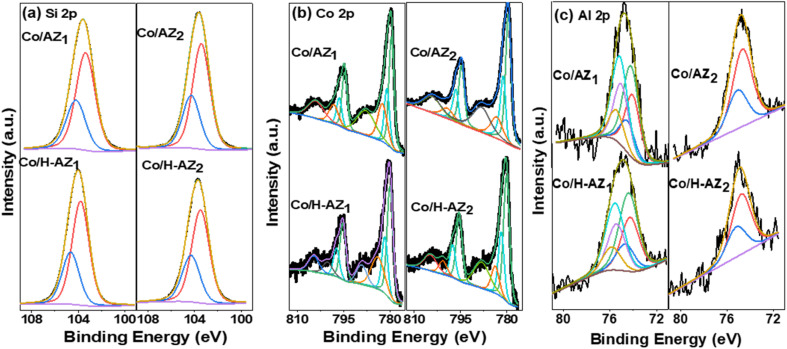
XPS of (a) Si 2p, (b) Co 2p and (c) Al 2p core level for Co/AZ_1_, Co/AZ_2_, Co/H-AZ_1_ and Co/H-AZ_2_.

X-ray photoelectron spectroscopy (XPS) was used to analyse the surface chemical composition of Co/AZ_1_, Co/AZ_2_ and Co/H-AZ_1_, Co/H-AZ_2_. The high-resolution of the Si 2p core levels of all the samples are shown in Fig. 6a. For the Co/H-AZ_1_ and Co/H-AZ_2_ sample, the Si 2p peak can be fitted with 2 Gaussian components at 103.8 and 104.6 eV which are deconvoluted into Si 2p_3/2_ and Si 2p_1/2_ with a spin–orbit splitting of 0.6 eV.

Comparatively, Si 2p appeared for Co/AZ_1_ and Co/AZ_2_ at 103.4 and 104.1 eV, respectively, and is associated with Si^4+^.^44^ Upon the introduction of H^+^ ions, the positions of these peaks were shifted towards higher binding energies as observed for Co/H-AZ_1_ and Co/H-AZ_2_ suggesting that H^+^ acts as an electron donor to Si, revealing the change in chemical environment. These shifts and changes are common when silica is functionalized with other elements to create absorbents or catalysts such as zeolites and clays.^45^ On the other hand, when the SiO_2_/Al_2_O_3_ mass ratio increased, there was no change meaning that the mass ratio did not affect Si 2p core level. The high-resolution Co 2p core levels were deconvoluted and several prominent peaks were noticed (Fig. 6b). The deconvolution for sample Co/AZ_1_, Co/AZ_2_ and Co/H-AZ_1_, Co/H-AZ_2_ Co 2p core levels unveiled peaks at 779.98 eV and 781.67 eV which belong to the Co 2p_3/2_ state of Co^3+^ and Co^2+^, respectively, while the peaks at 795.24 eV and 797.34 eV correspond to the Co 2p_1/2_. So, it can be noticed that the SiO_2_/Al_2_O_3_ mass ratio did not affect the Co 2p core level. More importantly, satellite peaks were detected at 782.81 and 803.38 eV. It is plausible that if the Co^3+^/Co^2+^ ratio is 2, cobalt exists in the Co_3_O_4_ phase. Finally, following deconvolution, two spin–orbit doublets (D1 and D2) emerged, indicating the coexistence of Co^2+^ and Co^3+^ according to XRD results as well.^46^ Furthermore, Fig. 6c depicts the high-resolution Al 2p core levels. The Al 2p photoemission peak for Co/AZ_1_ and Co/H-AZ_1_ can only be fitted to 2 peaks (fitted with a doublet with a spin–orbit splitting of 0.42 eV) peaking at 74.3 eV and 74.9 eV. This peak can be ascribed to Al in the +III oxidation state.^47^ However, when the SiO_2_/Al_2_O_3_ mass ratio increased, the Al 2p photoemission peak of Co/AZ_2_ and Co/H-AZ_2_ can be deconvoluted into several peaks corresponding to Al^3+^ existing in the bulk of the zeolite and Al^3+^ (Al–O–M) on the surface of the zeolite (Al–OH).^47^ Several prominent peaks could be explained by the fact that the chemical shift strongly depends on the SiO_2_/Al_2_O_3_ mass ratio and increases with increasing SiO_2_/Al_2_O_3_ ratios in the order O 1s > Si 2p > Na 1s > Al 2p.^48^ Dealumination must have led to the decrease in Si–O–Al proportion and, therefore increased the Si/Al ratio.^49,50^

### 3.6 Electrochemical analysis

**Fig. 7:**
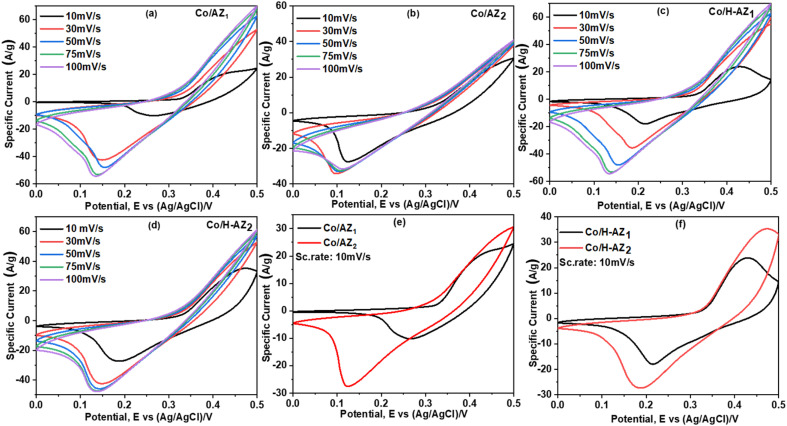
Electrocapacitive properties of different Co/AZ_1_, Co/AZ_2_, Co/H-AZ_1_ and Co/H-AZ_2_ electrode materials measured using a three-electrode system with 1 M KOH as aqueous electrolyte: CV curves for (a and b) Co/AZ_1_ and Co/AZ_2_ (c and d) Co/H-AZ_1_ and Co/H-AZ_2_ at different scan rates, (e and f) CV curves of Co/AZ_1_, Co/AZ_2_ and Co/H-AZ_1_, Co/H-AZ_2_ at a scan rate of 10 mV s^−1^.

**Fig. 8:**
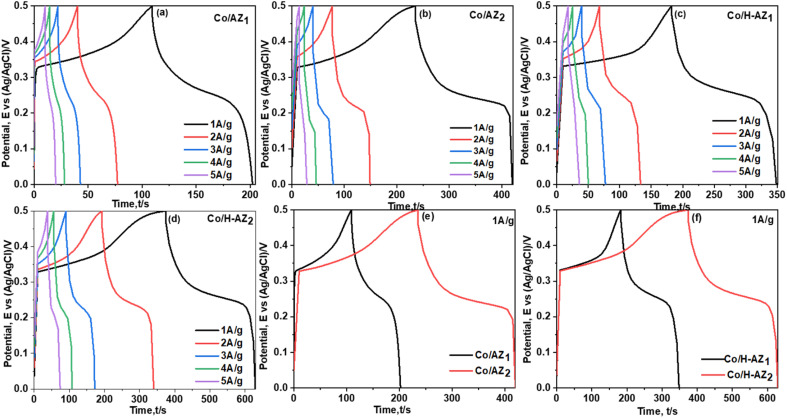
GCD curves for (a and b) Co/AZ_1_, Co/AZ_2_ and (c and d) Co/H-AZ_1_, Co/H-AZ_2_ at current densities ranging from 1 A g^−1^ to 5 A g^−1^, (e) GCD curves for Co/AZ_1_ Co/AZ_2_ at 1 A g^−1^, (f) GCD curves for Co/H-AZ_1_ Co/H-AZ_2_ at 1 A g^−1^.

**Fig. 9:**
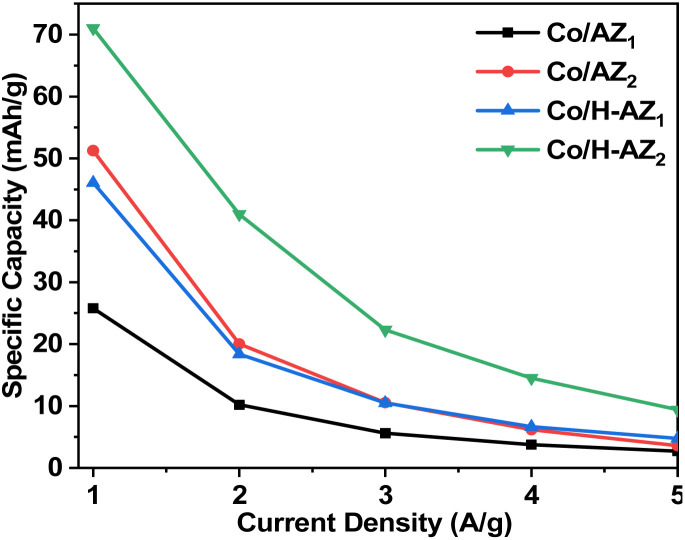
Specific capacity at various current densities for Co/AZ_1_, Co/AZ_2_, Co/H-AZ_1_ and Co/H-AZ_2_ electrode.

The electrochemical performance of the samples was investigated using cyclic voltammetry (CV), galvanostatic charge–discharge (GCD), electrochemical impedance spectroscopy (EIS) and linear sweep voltammetry (LSV) in a three-electrode system using 1.0 M KOH aqueous electrolyte. Fig. 7a–d display the CV curves of Co/AZ_1_, Co/AZ_2_ and Co/H-AZ_1_, Co/H-AZ_2_ measured at the scan rate of 10–100 mV s^−1^ in a potential window ranging from 0.0 to 0.5 V. For all the samples, typical redox peaks due to pseudocapacitive faradaic reactions of oxygen-derived functional groups in the porous structure were observed at 0.14 for all the samples. The observed polarization in the CV profiles for all samples suggests some difficulties in charge propagation, means there was no free ion insertion and ion diffusion at the low scan rate of 10 mV s^−1^ for all the samples. The behavior is attributed to faradaic hydrogen electrosorption occurs alongside electric double-layer charging therefore, induces non-symmetry CV profiles and separation of all the curves.^51^ Interestingly, the CV curves were distorted at higher scan rates as a result of weaker contributions from some pseudocapacitive species involved in the redox reactions at higher sweeping rates. It can be noticed that the SiO_2_/Al_2_O_3_ mass ratio increased with the surface area (Table 1), as well as the CV as Fig. 7e and f shows clearly, which is good for supercapacitor properties. Furthermore, EIS results (Fig. 11) corroborate this observation and also reveal that the Co/H-AZ_2_ electrode exhibited relatively smaller charge transfer resistance compared to the Co/AZ_2_ and comparatively, Co/AZ_1_ and Co/H-AZ_1_ which exhibited a bad charge transfer resistance when the SiO_2_/Al_2_O_3_ mass ratio increased. The Co/H-AZ_2_ and Co/AZ_2_ electrodes present a more vertical line in the low-frequency region compared with Co/AZ_1_ and Co/H-AZ_1_, indicating a better capacitive behavior.^52^Fig. 8 shows the galvanostatic charge/discharge (GCD) curves for all the samples in a potential window of 0 to 0.5 V at various current densities (1, 2, 3, 4 and 5 A g^−1^). It is evident that the discharge time of the Co/AZ_2_ and Co/H-AZ_2_ electrodes (mass ratio 2 SiO_2_/Al_2_O_3_) are much longer than that of its counterpart, thus, indicating that its specific capacity is much higher than that of the Co/AZ_1_ and Co/H-AZ_1_ electrodes (mass ratio 1 SiO_2_/Al_2_O_3_). This is also in agreement with the CV results discussed earlier. It can be noticed that when the mass ratio SiO_2_/Al_2_O_3_ increased, the time of charging and discharging increased which is good for supercapacitor properties. These GCD curves displayed non-linear shapes, thus, exposing the faradaic behaviour of both electrodes.^53^Fig. 9 displayed the specific capacity at different scan rates for all the samples. As can be observed, the specific capacity decreases with increasing scan rate. This general trend already reported in the literature is certainly due to the fact that when the current density increased, the charges did not have enough time to move through the pores during the charge/discharge process, leading to the low specific capacity.

From the results, it can be seen that the Co/H-AZ_2_ electrode exhibited a specific capacity of 72.5 mA h g^−1^ at 1 A g^−1^ which is much higher than that of 52.83 mA h g^−1^ at 1 A g^−1^ for the Co/AZ_2_ electrode. On the other hand, the Co/H-AZ_2_ electrode exhibited a specific capacity of 72.5 mA h g^−1^ at 1 A g^−1^ which is much higher than that of 45.97 F g^−1^ at 1 A g^−1^ for the Co/H-AZ_1_ electrode. So, when the SiO_2_/Al_2_O_3_ mass ratio increased, the specific capacity increased and this is in accordance with CV, GCD and EIS results. This may be due to the chemical stability of Co/H-AZ_2_ and Co/H-AZ_1_ as a result of the presence of H^+^ (ZSM5 has a high silicon to aluminium ratio). Whenever an Al^3+^ cation replaces a Si^4+^ cation, an additional positive charge is needed to keep the material charge neutral. This could be provided by H^+^, Na^+^.

**Fig. 10:**
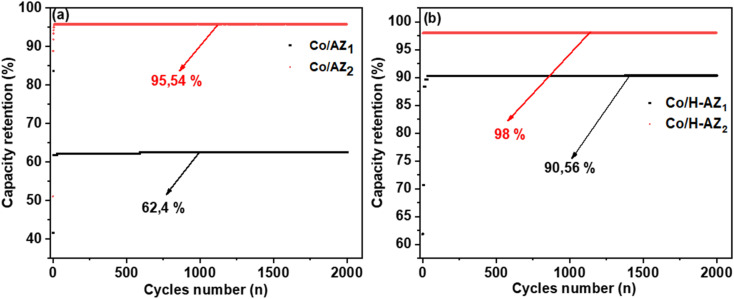
(a) Coulombic efficiency plot for Co/AZ_1_ and Co/AZ_2_, (b) Co/H-AZ_1_ and Co/H-AZ_2_ electrode.

Fig. 10a displays the coulombic efficiency for Co/AZ_1_ and Co/AZ_2_ electrodes with the current density at 5 A g^−1^. Interestingly, the Co/AZ_2_ coulombic efficiency was 95.54% after 2000 cycles compared to Co/AZ_1_ electrode which was 62.4%. Fig. 10b shows coulombic efficiency for Co/H-AZ_2_ and Co/H-AZ_1_ electrodes at 5 A g^−1^. Interestingly, the Co/H-AZ_2_ electrode coulombic efficiency was 98% after 2000 cycles and Co/H-AZ_1_ was 90.56%. This is also proof that despite the presence of the proton H^+^ which improves the electrochemical performance with increasing of SiO_2_/Al_2_O_3_ mass ratio. The higher value of the capacity retention suggests that the electrode might be stable for a longer period. This result is also in good agreement with those of CV, GCD and EIS, which clearly shows that when the SiO_2_/Al_2_O_3_ mass ratio increased, the supercapacitive performance increased as well.

**Fig. 11:**
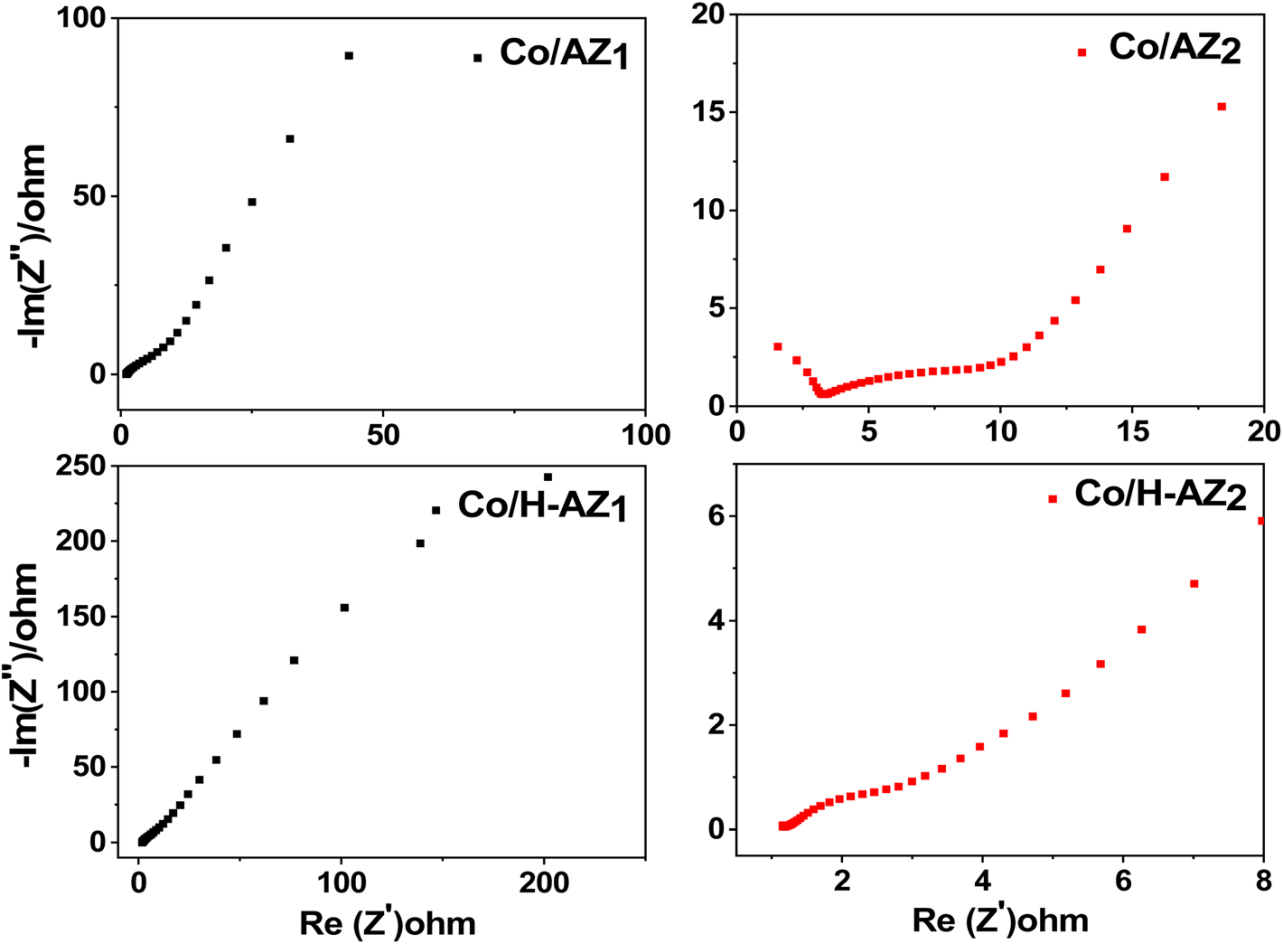
EIS curves of Co/AZ_1_, Co/AZ_2_, Co/H-AZ_1_, Co/H-AZ_2_.

The specific capacity of Co/AZ_1_, Co/AZ_2_ and Co/H-AZ_1_, Co/H-AZ_2_ were calculated from GCD curves at 1 A g^−1^ using eqn (1) and (2),^54^ (where *C*_s_ is the specific capacitance (F g^−1^), *Q*_s_ is the specific capacity (mA h g^−1^), *I* is the constant discharge current (A), Δ*t* is the discharge time (s), Δ*V* is the potential window (V) and *m* is the mass of the sample in the electrode (g))^55^1
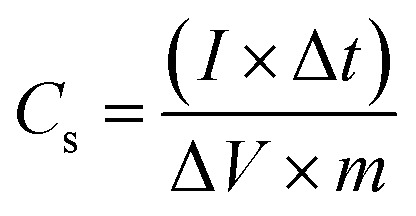
2
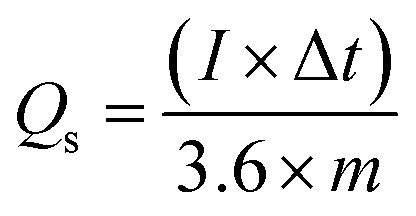


From Table 1, sample Co/AZ_2_ possesses the highest specific surface area as compared to Co/AZ_1_ and the same applies to sample Co/H-AZ_2_ and Co/H-AZ_1_. It is widely reported that an electrode material with a high specific surface area very often yields high supercapacitive performance.^41,42^ Inherently, the present work reveals that Co/H-AZ_1_ and Co/H-AZ_2_ exhibit a high specific surface area relative to their counterparts and their electrodes displayed a higher specific capacity in comparison with its counterpart. This observation implies that the specific surface area is not the only factor that contributes to the overall electrochemical performance, there is also the effect of the SiO_2_/Al_2_O_3_ ratio which may also play a role.

**Fig. 12:**
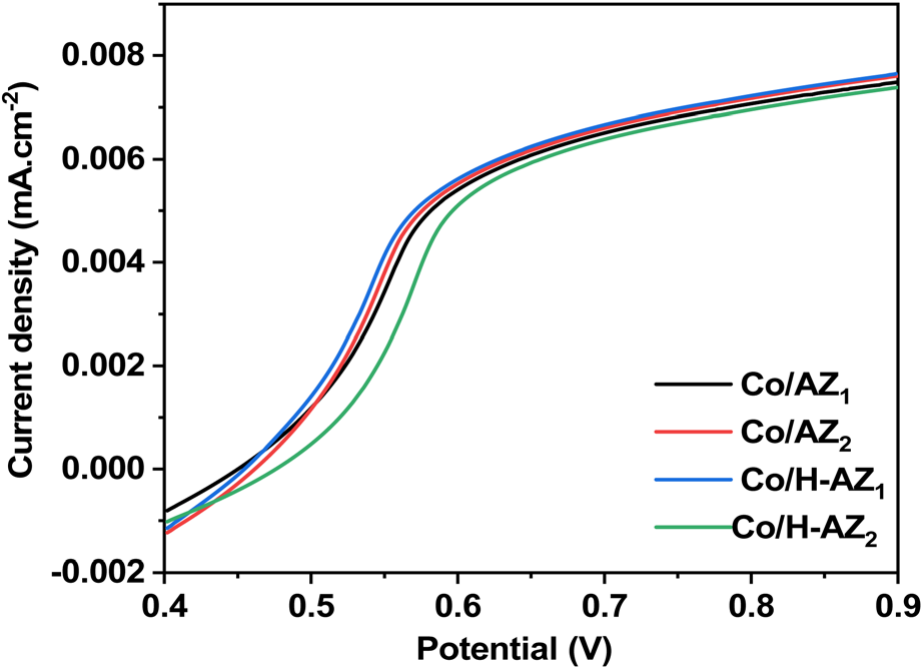
The linear sweep voltammetry curves of Co/AZ_1_, Co/AZ_2_, Co/H-AZ_1_ and Co/H-AZ_2_.

The linear sweep voltammetry curves of Co/AZ_1_, Co/AZ_2_, Co/H-AZ_1_ and Co/H-AZ_2_ electrodes are shown in Fig. 12 which is a typical LSV plot for a non-diffusion limited redox system. Co/AZ_1_, Co/AZ_2_, Co/H-AZ_1_ show their activity with half-wave potentials (*E*_1/2_ = *E*_2/2_ = *E*_3/3_ = 0.525) V and current density (*J*_L_ = 0.0020 mA cm^−2^). Co/H-AZ_2_ shows its activity with half-wave potential (*E*_4/2_ = 0.550 V) and current density (*J*_L_ = 0.0025 mA cm^−2^). So we can conclude that among these results, the best result is obtained for Co/H-AZ_2_ (mass ratio 2: 8.3) compared to Co/H-AZ_1_ (mass ratio 1: 6.3).^56,57^ It can be seen that the proton H^+^ and the SiO_2_/Al_2_O_3_ mass ratio have played a significant role.

## 4. Conclusions

In summary, the present work shows that we have synthesized successfully amorphous zeolites loaded with cobalt oxide at two different SiO_2_/Al_2_O_3_ mass ratios *via* the steam-assisted crystallization method for supercapacitor electrode application. It was demonstrated that when the mass ratio SiO_2_/Al_2_O_3_ (8.3) increased, the super capacitive performance increased and LSV also shows a good result. The impregnation of cobalt oxide on amorphous zeolites H-AZ_1_, AZ_1,_ H-AZ_2_, AZ_2_, used as electrodes, were observed to significantly enhance the electrochemical performance. The specific capacity (*C*_s_) value obtained for Co/H-AZ_2_ was about 72.5 mA h g^−1^ compared to that of Co/H-AZ_1_ (45.97). The specific capacity (*C*_s_) value obtained for Co/AZ_2_ was about 52.83 mA h g^−1^ compared to that of Co/AZ_1_ (26). It was clearly shown that Co/H-AZ_2_ and Co/H-AZ_1_ exhibited an excellent coulombic efficiency of 98% and 90.56% respectively, after 2000 cycles from GCD. Although improved electrochemical performance is attributed to the effect of H^+^ ions in H-AZ_1_ and H-AZ_2_ zeolites and the optimal faradaic nature, it can be noticed that when the SiO_2_/Al_2_O_3_ mass ratio increased, the supercapacitor properties increased as well. Finally, it has been demonstrated in this work that Co/H-AZ_2_ with a high ratio SiO_2_/Al_2_O_3_ ratio (8.3) has a good super capacitive performance owing to the synergistic effect of H^+^ ions at the electrode/electrolyte interface. This work sheds new light on the improvement of the electrochemical performance of zeolite based supercapacitors precisely through the effect of the SiO_2_/Al_2_O_3_ mass ratios.

## Author contributions

Saureille Ngouana Moafor contributed to conceptualizing, the experiment, analysis and writing of the manuscript. Linda Jewell and Lambi John Ngolui have contributed to the supervision. All the others co-authors have discussed the results and commented on the manuscript.

## Conflicts of interest

There are no conflicts of interest to declare.

## Supplementary Material
